# Intraoperative Fentanyl Consumption Does Not Impact Cancer Recurrence or Overall Survival after Curative Colorectal Cancer Resection

**DOI:** 10.1038/s41598-017-11460-1

**Published:** 2017-09-07

**Authors:** Ying-Hsuan Tai, Hsiang-Ling Wu, Wen-Kuei Chang, Mei-Yung Tsou, Hsiu-Hsi Chen, Kuang-Yi Chang

**Affiliations:** 1 Department of Surgery, Taipei Veterans General Hospital, Yuanshan Branch, Yilan, Taiwan; 2 Department of Anesthesiology, Taipei Veterans General Hospital, Taipei, Taiwan; 3 Division of Biostatistics, Graduate Institute of Epidemiology and Preventive Medicine, College of Public Health, National Taiwan University, Taipei, Taiwan; 4 School of Medicine, National Yang-Ming University, Taipei, Taiwan

## Abstract

Whether opioid use in cancer surgery would promote tumor dissemination in humans is inconclusive. We investigated the effect of intraoperative fentanyl dose on colorectal cancer (CRC) prognosis following resection in this retrospective study. A total of 1679 patients with stage I-III CRC undergoing tumor resection between January 2011 and December 2014 were evaluated through August 2016. Postoperative recurrence-free survival (RFS) and overall survival (OS) were analyzed using Cox regression models. Multivariable Cox regression analysis demonstrated no dose-response association between the amount of fentanyl dose and RFS (adjusted hazard ratio: 1.03, 95% CI: 0.89–1.19) or OS (adjusted hazard ratio: 0.84, 95% CI: 0.64–1.09). Patients were further classified into the high- and low-dose groups by the median of fentanyl dose (3.0 μg·kg^−1^), and there was no significant difference in RFS or OS between groups, either (adjusted hazard ratio: 0.93, 95% CI: 0.74–1.17 for RFS; 0.79, 95% CI: 0.52–1.19 for OS). We concluded that intraoperative fentanyl consumption has no impact on recurrence-free or overall survival in patients after curative CRC resection.

## Introduction

Opioids have long been the mainstay of analgesics for cancer-related pain and perioperative acute pain. However, it has been established that opioids inhibit cellular and humoral immune function in humans^1, 2^. Although there are no data directly implicating opioids in cancer genesis in humans, animal models strongly suggest they may contribute to cancer recurrence in the clinical setting^3^. This tumor-promoting property was also demonstrated in fentanyl^2, 4^. Nonopioid analgesia helps to preserve the function of natural killer (NK) cell, the primary defense against cancer cells, and reduces metastatic spread of cancer in animals^5^.

It has been proposed that opioids exert their effects on tumor growth by activation of the mu-opioid receptor (MOR). Overexpression of the MOR in human non–small cell lung cancer (NSCLC) was suggested to promote tumor growth and progression^6^. Higher MOR expression and greater opioid requirement are associated with poor oncologic outcomes in patients with metastatic prostate cancer^7^. MORs have also been demonstrated in the nuclei of human colon cancer cells, and exposure of these cells to morphine increased secretion of urokinase plasminogen activator, a promoter of tumor metastasis^8^.

Since opioids are common analgesics administered during and after cancer surgery, it is of great importance to evaluate their effects on oncologic outcomes in clinical setting. We conducted this retrospective cohort study in patients following CRC surgery to analyze the associations between intraoperative fentanyl dose and cancer recurrence or overall mortality using multivariable Cox regression models. Dose-response associations between the amount of fentanyl dose and cancer outcomes were further evaluated and major prognostic predictors of CRC outcomes were also considered in the analysis to reduce potentially confounding effects^9–11^.

## Methods

### Setting and patient selection

**Figure 1:**
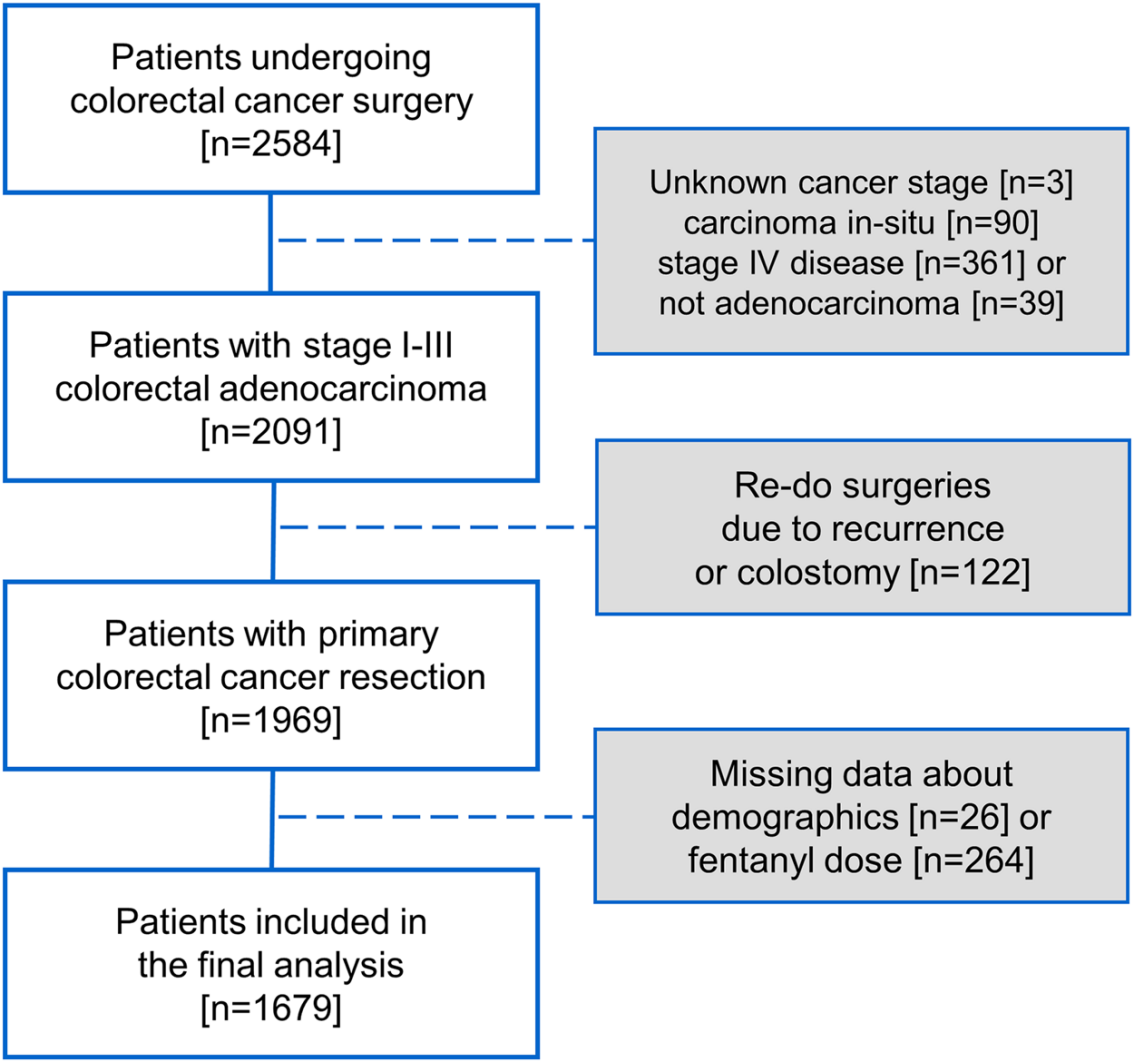
Flow diagram for patient inclusion.

The study was approved by the Institutional Review Board of Taipei Veterans General Hospital, Taipei, Taiwan (IRB-TPEVGH No. 2015-11-010CC). Written informed consent was waived, and all the study materials were anonymized and de-identified before analysis. We utilized the electronic medical database to identify all the patients with CRC undergoing curative resection of primary cancer at the Taipei Veterans General Hospital, Taipei, Taiwan, between January 2011 and December 2014. There was no significant alteration in clinical practice at the centre in the time period of the study. We excluded patients with carcinoma *in-situ* or stage IV disease determined at the time of surgery, unknown cancer stage, pathology other than adenocarcinoma, re-do surgeries due to recurrence or colostomy, or missing data about demographics or fentanyl dose (Fig. 1).

### Anesthetic management

All patients undergoing major abdominal surgery at our hospital were given balanced general anesthesia, typically inducted with fentanyl 1–2 μg·kg^−1^ and propofol 1–2 mg·kg^−1^, and neuromuscular antagonism with rocuronium 0.8 mg·kg^−1^ or cisatracurium 0.2 mg·kg^−1^. Anesthesia was maintained with sevoflurane 2–3 vol% or desflurane 6–8 vol% in oxygen, with a fraction of inspired oxygen of 40 to 60% at the anesthetist’s discretion.

Fentanyl was the mainstay narcotics used during cancer surgery at this centre. In addition to anesthetic induction, an intravenous bolus of fentanyl 50 μg may be administered before surgical incision if patients did not receive epidural analgesia. If uncontrolled pain was suspected based on hemodynamic changes (e.g. tachycardia and hypertension), the anesthetist may escalate the concentration of volatile anesthetics. Alternatively, single or consecutive intravenous doses of fentanyl would be administered to relieve pain at the anesthetist’s discretion. When an epidural was used, the typical regimen included a loading dose of local anesthetics (lidocaine 1% or 2%) with or without fentanyl 50 μg, followed by an continuous infusion of local anesthetics (bupivacaine 0.25% or 0.5%) with fentanyl 5 μg·ml^−1^ at a rate of 5–10 ml·hour^−1^.

Patients would have regular postoperative surveillance at our hospital if they would be eligible for curative-intent surgery. Carcinoembryonic antigen (CEA) was tested every 3 to 6 months for 2 years, then every 6 months for 3 years. Abdomen and chest computed tomography (CT) scans were performed for colon cancer every 6 to 12 months for up to 5 years for patients at high risk of recurrence. For rectal cancer, pelvis CT was added every 3 to 6 months for 2 years, then every 6 to 12 months for up to 5 years for those at high risk of recurrence. Colonoscopy surveillance was conducted at 1 year and repeated 1 to 3 years thereafter.

An electronic medical database was used to determine the baseline characteristics and risk factors for cancer recurrence and mortality, including demographics, pre-treatment CEA level^12^, perioperative packed red blood cell (pRBC) transfusion^13^, pathologic findings (tumor differentiation, mucinous or signet-ring histology^9^, lymphovascular invasion^10^, and perineural invasion^11^); whether preoperative or postoperative adjuvant chemotherapy or radiotherapy was used. Current status of each patient was determined by documentation of follow-up visits to the hospital’s outpatient clinic or subsequent admissions. Tumor nodes metastasis (TNM) staging was obtained from the pathologic reports and translated into stages I to III according to the American Joint Committee on Cancer criteria (AJCC-7 staging system)^14^. Tumor location was divided into right-sided tumor (cecal to splenic flexure) and left-sided colon (splenic flexure to rectum). Adjuvant therapy given in the form of chemotherapy (leucovori and oxaliplatin or fluorouracil, capecitabine, tegafur-uracil for stage II or III disease) or radiotherapy was at the discretion of surgeons and patients. Any adjuvant therapy was defined as given within 90 days of surgery. Cancer recurrence was determined by imaging examinations (CT, magnetic resonance imaging, sonography, bone scan, or plain radiogram) and defined according to response evaluation criteria in solid tumours (RECIST) guidelines^15^.

Medical records of all the patients included were reviewed and data extracted by research assistants who were not involved in the data analysis. Specialist anesthetists assisted them with data extraction when necessary. The quality of the extracted data was verified through random sampling by the authors. Data were collected up to the end of August 2016.

The primary endpoint was recurrence-free survival (RFS), which was defined as time from the date of surgery to the date of cancer recurrence. The secondary endpoint was overall survival (OS), defined as time from the date of surgery to the date of death. For those without the event of cancer recurrence or death, their survival times are regarded as the corresponding censored observations.

### Data analysis and statistics

Patient characteristics, surgical data and pathologic findings were compared between groups using t tests, Mann-Whitney U tests and chi square tests as appropriate. The Kaplan–Meier method was used to calculate the recurrence-free and overall survival of patients from the date of surgery to the date of detection of cancer recurrence and death, respectively; patients without recurrence or alive were censored in the corresponding survival analyses at the last observed day or end of follow-up time (August 31, 2016). Cox proportional hazards regression models were used to evaluate the correlation between the exposure amount of fentanyl and risks of cancer recurrence or overall mortality in the univariate analysis. Multivariable models were applied to adjust other independent risk factors obtained from forward model selection processes with an entry criterion of 0.05. Furthermore, we used the median of fentanyl dose (3.0 μg·kg^−1^) to define the high-dose (>3.0 μg·kg^−1^) and low-dose (<3.0 μg·kg^−1^) groups. Fentanyl dose was regarded as a categorical variable in the univariate and multivariable analyses as well. A two-sided significance level of 0.05 was used to assess statistically significant difference. All the statistical analyses were conducted with IBM SPSS Statistics, Version 23.0 (Armonk, NY: IBM Corp.).

## Results

A total of 1679 patients were selected for further analyses after the exclusion processes (Fig. 1). The minimum and maximum fentanyl dosages were 0.2 and 7.8 μg·kg^−1^, respectively, and the interquartile range of fentanyl dose was from 2.5 to 3.5 μg·kg^−1^. The median of fentanyl dose administered intraoperatively was 3.0 μg·kg^−1^ and this number was subsequently utilized to separating patients into the high- and low-dose groups. The differences in mean fentanyl dose between the two groups were 1.24 μg·kg^−1^ (*p* < 0.001). The high-dose group was more likely to be female and have epidural blocks, neoadjuvant chemotherapy or radiotherapy, perioperative pRBC transfusion, lower body height and weight and longer follow-up intervals (Table 1).

**Table 1:** Patient demographics.

	Low-dose (N = 877)	High-dose (N = 802)	*p*	Total (N = 1679)
Fentanyl dose, μg·kg^−1^	2.45 ± 0.42	3.69 ± 0.61	<0.001	3.04 ± 0.81
Age, year	68 ± 14	67 ± 14	0.135	68 ± 14
Gender, male	591 (67.4%)	426 (53.1%)	<0.001	1017 (60.6%)
ASA ≧ 3	319 (36.4%)	283 (35.3%)	0.643	602 (35.9%)
Body height, cm	163 ± 8	159 ± 9	<0.001	161 ± 9
Body weight, kg	67.0 ± 11.7	57.5 ± 10.5	<0.001	62.5 ± 12.1
**Comorbidites**				
Diabetes	223 (25.4%)	184 (22.9%)	0.235	407 (24.2%)
Coronary artery disease	91 (10.4%)	76 (9.5%)	0.538	167 (9.9%)
Heart failure	62 (7.1%)	54 (6.7%)	0.786	116 (6.9%)
Stroke	58 (6.6%)	54 (6.7%)	0.922	112 (6.7%)
Chronic kidney disease	125 (14.3%)	103 (12.8%)	0.399	228 (13.6%)
Pretreatment CEA, μg·L^−1^	2.9 (2.0–6.3)	3.0 (1.9–6.3)	0.896	3.0 (1.9 -6.3)
Tumour location			0.417	
Right-sided tumour	640 (73.0%)	571 (71.2%)		1211 (72.1%)
Left-sided tumour	237 (27.0%)	231 (28.8%)		468 (27.9%)
Laparoscopic surgery	105 (12.0%)	91 (11.3%)	0.690	196 (11.7%)
Epidural block	18 (2.1%)	41 (5.1%)	0.001	59 (3.5%)
Anaesthesia time, min	285 (240–345)	300 (240–360)	0.461	300 (240–345)
AJCC stage			0.543	
Stage I	231 (26.3%)	212 (26.4%)		443 (26.4%)
Stage II	351 (40.0%)	302 (37.7%)		653 (38.9%)
Stage III	295 (33.6%)	288 (35.9%)		583 (34.7%)
**Pathologic features**				
Tumour differentiation			0.102	
Good	53 (6.1%)	56 (7.0%)		109 (6.5%)
Moderate	752 (85.9%)	698 (87.0%)		1450 (86.5%)
Poor	70 (8.0%)	48 (6.0%)		118 (7.0%)
Mucinous histology	42 (4.8%)	33 (4.1%)	0.498	75 (4.5%)
Signet-ring histology	28 (3.2%)	31 (3.9%)	0.460	59 (3.5%)
Lymphovascular invasion	221 (25.3%)	181 (22.6%)	0.198	402 (24.0%)
Perineural invasion	104 (11.9%)	90 (11.2%)	0.671	194 (11.6%)
pRBC transfusion	158 (18.0%)	213 (26.6%)	<0.001	153 (9.1%)
Preoperative C/T ± R/T	60 (6.8%)	93 (11.6%)	0.001	770 (45.9%)
Postoperative C/T	406 (46.3%)	364 (45.4%)	0.709	20 (1.2%)
Postoperative R/T	12 (1.4%)	8 (1.0%)	0.484	75 (4.5%)
Follow-up time, months	29.86 (20.67–44.48)	34.04 (20.9–48.49)	0.007	31.54 (20.76–46.62)

Values were mean ± SD, counts (percent), or median (interquartile range). Continuous variables are analysed with Wilcoxon rank-sum tests; categorical variables are analysed with Pearson chi-square tests or Mann-Whitney U tests, as appropriate. BMI: body mass index; ASA physical status: American Society of Anesthesiologists physical status; CEA: carcinoembryonic antigen; AJCC: American Joint Committee on Cancer; pRBC: packed red blood cell; C/T: chemotherapy; R/T: radiotherapy.

### The association between fentanyl dose and recurrence-free survival

**Figure 2:**
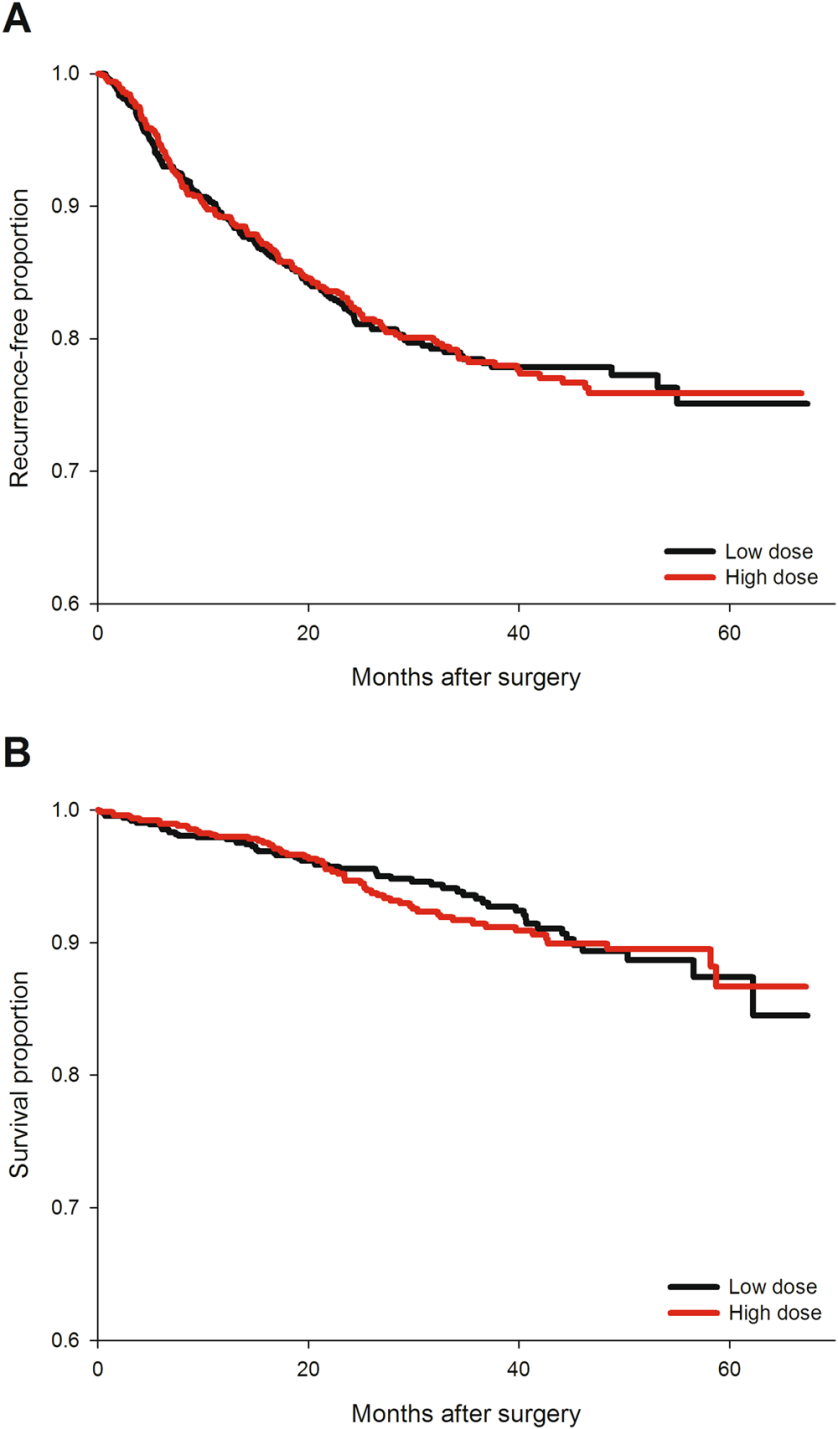
Kaplan–Meier curves for recurrence-free and overall survival of high- and low-dose groups. No significant difference in recurrence-free survival (Fig. 2A) or overall survival (Fig. 2B) after surgery was noted when comparing high- and low-dose groups.

No dose-response relationship was noted between the amount of fentanyl dose and RFS in the univariate analysis. No significant difference in RFS was noted between high- and low-dose groups, either (*p* = 1.00 by log-rank test, Fig. 2A). Univariate analysis revealed several significant risk factors of cancer recurrence (Table 2), including chronic kidney disease, higher pretreatment CEA level, longer anesthesia time, perioperative pRBC transfusion, advanced cancer stage, specific pathologic findings (poor differentiation, signet-ring histology, lymphovascular invasion, and perineural invasion), preoperative chemotherapy and/or radiotherapy, and postoperative chemotherapy or radiotherapy. After the model selection, eight independent prognostic factors for RFS were identified, including chronic kidney disease (HR = 1.53), preoperative CEA level (on base-10 logarithmic scale, HR = 1.73), cancer stage (II vs. I, HR = 2.78; III vs. I, HR = 6.77), perioperative pRBC transfusion (HR = 1.39), pathologic lymphovascular invasion (HR = 1.37) and perineural invasion (HR = 1.68), preoperative chemotherapy and/or radiotherapy (HR = 2.46), and postoperative radiotherapy (HR = 1.95) (Table 3). After taking these significant predictors into account, the effect of fentanyl dose on RFS after resection surgery was non-significant in the multivariable model, either as a linear (adjusted HR = 1.03, 95% CI = 0.89–1.19) or a categorical variable (adjusted HR = 0.93, 95% CI = 0.74–1.17).

**Table 2:** Univariate analysis of cancer recurrence and all-cause mortality.

	Cancer recurrence			All-cause mortality		
	HR	95% C.I.	*p*	HR	95% C.I.	*p*
Fentanyl dose (linear)	1.06	0.92–1.21	0.440	1.05	0.84–1.31	0.689
Fentanyl dose (categorical)	1.00	0.80–1.25	1.00	1.05	0.73–1.51	0.774
Age	1.01	1.00–1.02	0.053	1.05	1.03–1.06	<0.001
Gender (F vs. M)	1.03	0.82–1.29	0.802	1.20	0.82–1.74	0.349
Body height	1.00	0.99–1.01	0.964	0.99	0.97–1.01	0.205
Body weight	0.99	0.98–1.00	0.153	0.97	0.95–0.99	<0.001
ASA ≥ 3	1.22	0.97–1.54	0.087	2.87	1.99–4.14	<0.001
Diabetes	1.13	0.87–1.46	0.358	1.91	1.31–2.78	0.001
Coronary arterial disease	1.17	0.82–1.67	0.387	1.99	1.23–3.22	0.005
Heart failure	1.03	0.66–1.60	0.900	2.49	1.49–4.16	0.001
Stroke	0.94	0.57–1.53	0.794	2.34	1.34–4.09	0.003
Chronic kidney disease	1.65	1.24–2.20	0.001	2.94	1.97–4.37	<0.001
Pretreatment CEA*	2.65	2.22–3.16	<0.001	2.42	1.82–3.22	<0.001
Laparoscopy surgery	0.92	0.64–1.31	0.649	0.97	0.56–1.70	0.920
pRBC transfusion	1.93	1.52–2.45	<0.001	3.79	2.64–5.44	<0.001
Epidural block	0.97	0.54–1.73	0.916	0.55	0.17–1.73	0.304
Anaesthesia time**	1.54	1.15–2.06	0.004	2.31	1.44–3.70	0.001
Preoperative C/T ± R/T	2.03	1.50–2.75	<0.001	1.56	0.92–2.64	0.099
Postoperative C/T	3.10	2.41–3.97	<0.001	1.33	0.92–1.91	0.129
Postoperative R/T	4.16	2.28–7.61	<0.001	3.74	1.53–9.16	0.004
Left- vs. right-sided tumour	0.90	0.70–1.16	0.416	1.22	0.82–1.80	0.322
AJCC Stage			<0.001			<0.001
Stage II vs. I	3.56	2.11–5.99	<0.001	3.23	1.57–6.62	0.001
Stage III vs. I	10.91	6.65–17.89	<0.001	5.49	2.73–11.01	<0.001
Tumour differentiation			<0.001			0.029
Moderate vs. good	2.18	1.16–4.10	0.016	2.86	0.91–9.01	0.073
Poor vs. good	4.14	2.05–8.36	<0.001	5.08	1.43–18.01	0.012
Mucinous histology	1.27	0.77–2.10	0.355	2.16	1.13–4.14	0.020
Signet-ring histology	1.89	1.18–3.05	0.009	2.13	1.04–4.36	0.039
Lymphovascular invasion	2.71	2.16–3.39	<0.001	2.24	1.55–3.24	<0.001
Perineural invasion	3.09	2.40–3.99	<0.001	2.30	1.50–3.54	<0.001

Fentanyl dose is considered as a linear predictor or categorical variable (<3.0 μg·kg^−1^ or >3.0 μg·kg^−1^) in the univariate analysis. HR: hazard ratio; F: female, M: male; BMI: body mass index; ASA physical status: American Society of Anesthesiologists physical status; CEA: carcinoembryonic antigen; pRBC: packed red blood cell; C/T: chemotherapy; R/T: radiotherapy; AJCC: American Joint Committee on Cancer. *On base-10 logarithmic scale; **On base-2 logarithmic scale.

**Table 3:** Forward model selection for recurrence-free survival.

	**HR**	**95% C**.**I**.	***p***
Chronic kidney disease	1.53	1.13–2.08	0.006
Pretreatment CEA*	1.73	1.43–2.09	<0.001
Preoperative C/T ± R/T	2.46	1.79–3.38	<0.001
Postoperative R/T	1.95	1.02–3.74	0.043
Stage			<0.001
II vs. I	2.78	1.64–4.71	<0.001
III vs. I	6.77	4.03–11.35	<0.001
Lymphovascular invasion	1.37	1.05–1.78	0.018
Perineural invasion	1.68	1.27–2.22	<0.001
pRBC Transfusion	1.39	1.08–1.81	0.012
Fentanyl dose (linear)	1.03	0.89–1.19	0.732
Fentanyl dose (categorical)	0.93	0.74–1.17	0.560

Fentanyl dose is considered as a linear predictor or categorical variable (<3.0 μg·kg^−1^ or >3.0 μg·kg^−1^) in the multivariable analysis. HR: hazard ratio; CEA: carcinoembryonic antigen; C/T: chemotherapy; R/T: radiotherapy; pRBC: packed red blood cell. *On base-10 logarithmic scale.

### The association between fentanyl dose and overall survival

No dose-dependent association was identified between the amount of fentanyl dose and OS in the univariate analysis. The difference in OS after surgery was not significant between the high- and low-dose groups, either (*p* = 0.77, Fig. 2B). In the univariate analysis, variables associated with shorter survival were older age, ASA physical status >3, lower body weight, comorbidities (diabetes, ischemic heart disease, heart failure, old stroke, and chronic kidney disease), higher pretreatment CEA level, longer anesthesia time, perioperative pRBC transfusion, advanced cancer stage, specific pathologic findings (poor differentiation, mucinous and signet-ring histology, lymphovascular invasion, and perineural invasion) and postoperative radiotherapy (Table 2). Nine independent prognostic determinants for OS were identified after multivariable analysis (Table 4), including older age (HR = 1.03), lower body weight (HR = 1.02), chronic kidney disease (HR = 1.74), higher pretreatment CEA (on base-10 logarithmic scale, HR = 1.53), longer anesthesia time (on base-2 logarithmic scale, HR = 2.33), perioperative pRBC transfusion (HR = 2.17), cancer stage (II vs. I, HR = 1.87; III vs. I, HR = 3.01), signet-ring histology (HR = 2.41), and pathologic perineural invasion (HR = 1.83). Adjusting for covariates, no association between fantanyl dose and overall mortality after surgery was noted, either as a linear (adjusted HR = 0.84, 95% CI = 0.64–1.09) or a categorical variable (adjusted HR = 0.79, 95% CI = 0.52–1.19).

**Table 4:** Forward model selection for overall survival.

	HR	95% C.I.	*p*
Age	1.03	1.01–1.05	<0.001
Body weight	0.98	0.96–1.00	0.020
Chronic kidney disease	1.74	1.12–2.69	0.014
Pretreatment CEA*	1.53	1.12–2.11	0.008
Anaesthesia time**	2.33	1.43–3.78	0.001
Stage			0.004
II vs. I	1.87	0.90–3.89	0.096
III vs. I	3.01	1.45–6.25	0.003
Signet-ring histology	2.41	1.15–5.03	0.019
Perineural invasion	1.83	1.16–2.88	0.009
pRBC transfusion	2.17	1.46–3.22	<0.001
Fentanyl dose (linear)	0.84	0.64–1.09	0.188
Fentanyl dose (categorical)	0.79	0.52–1.19	0.258

Fentanyl dose is considered as a linear predictor or categorical variable (<3.0 μg·kg^−1^ or >3.0 μg·kg^−1^) in the multivariable analysis. HR: hazard ratio; CEA: carcinoembryonic antigen; pRBC: packed red blood cell. *On base-10 logarithmic scale; **On base-2 logarithmic scale.

## Discussion

This study showed no definite association between intraoperative fentanyl dose and oncologic outcomes in stage I-III CRC patients after surgical resection. Despite the contradiction to the findings of some prior investigations, our study provided new evidence to reject the hypothetical relationships between intraoperative fentanyl dose and CRC prognosis after surgery with two strengths. We regarded the amount of fentanyl dose as a linear predictor to evaluate the dose-response association between CRC cancer outcomes and intraoperative fentanyl dose. Furthermore, compared with previous studies, we collected comprehensive information about important clinicopathologic predictors (e.g. pre-treatment CEA level and pathologic lymphovascular invasion) to minimize confounding effects of critical prognostic factors.

Intraoperative administration of sufentanil has been reported to be associated with shorter biochemical-free recurrence survival in patients with prostate cancer^16^. Large opioid requirement and high expression of the MOR were associated with worse progression-free survival and overall survival in patients with metastatic prostate cancer^7^. Higher opioid consumption during surgery has been reported to be a risk factor for overall survival in patients with stage I but not stage II-III NSCLC^17^. Maher and colleagues reported an association between increased opioid doses during initial 96-hours postoperative period and higher recurrence rate of NSCLC within 5 years^18^. However, they found no difference in intraoperative opioid administration among those with or without recurrence of NSCLC at the 5 year follow-up. Similarly, the association between the intraoperative administration of sufentanil and higher rate of recurrence after breast cancer surgery was not significant^19^. Presumably narcotics played little role in postoperative cancer outcomes but factors increasing narcotic demand (e.g. more aggressive tumor and more extensive surgical resection) affected recurrence and survival after cancer surgery.

Although most clinically used opioids exert their analgesic effects through MOR, different mu opioids may have varying effects on immune function^20^. Beilin and co-workers showed patients receiving high-dose fentanyl (75–100 µg·kg^−1^) had more prolonged suppression of NK cell than those receiving low-dose fentanyl (up to 6 µg·kg^−1^)^2^. Nevertheless, our result did not suggest the tumour-promoting effect of fentanyl in the perioperative period. Additionally, the effect of morphine on tumor growth is mixed and conflicting in previous literature^21^. Morphine was demonstrated to suppress cell-mediated immunity in healthy volunteers, including NK cell cytotoxicity^1^. Morphine at clinically relevant doses increases angiogenesis and promotes breast tumor growth in mice^3^. However, administration of morphine in rats undergoing laparotomy attenuated a surgery-induced increase in tumor retention^22^. Tramadol stimulates NK cell activity, both in rodents and humans and can block the enhancement of lung metastasis induced by surgery and prevent the surgery-induced suppression of NK cell activity in a rat model^23^.

Several retrospective clinical studies revealed the association between the expression of MOR and tumor progression in specific cancer types, including breast, prostate, lung, and esophageal squamous cell carcinoma^24, 25^. Although there was no report focused on the effect of MOR on cancer outcomes in CRC, a preclinical study suggested the metastasis-promoting potential of opioids in colorectal tumor cells^8^. It has been proposed mu-opioids stimulate tumor proliferation and invasion through proangiogenic effects^3^, and angiogenesis is also found to be an important mechanism in tumor growth and invasion in CRC^26–30^. Our observation that fentanyl dose does not influence recurrence rate contrasts with some previous studies. Apart from the difference in cancer types, the discrepancy may come from the relatively small doses of opioids used in our subjects compared with previous studies. Whether there exists a threshold dose of opioids to produce tumor-promoting effects in human remains to be seen.

We would like to acknowledge the limitations of the present investigation. First, this is a retrospective cohort study and not a randomized controlled trial. Although many important prognostic factors had been considered in this study, it is difficult to evaluate potential effects from unmeasured confounders. Second, the total amount of opioids administered postoperatively was not available for analysis due to the limitations of databank. Third, we did not further assess the effect of non-opioid analgesics that might interact with opioids to alter immune responses which may affect oncologic outcomes after surgery by reason of data availability.

## Conclusions

Intraoperative fentanyl consumption has no impact on cancer recurrence or overall survival in patients after curative colorectal cancer resection. Our findings provided new evidence to reject the association between intraoperative fentanyl dose and long-term outcomes after colorectal cancer surgery. Further prospective studies are needed to elucidate the relationship between intraoperative opioid use and cancer prognosis.
